# The possible role of gut microbiota dysbiosis in the pathophysiology of delirium in older persons

**DOI:** 10.20517/mrr.2023.15

**Published:** 2023-05-26

**Authors:** Andrea Ticinesi, Alberto Parise, Antonio Nouvenne, Nicoletta Cerundolo, Beatrice Prati, Tiziana Meschi

**Affiliations:** ^1^Microbiome Research Hub, University of Parma, Parma 43124, Italy.; ^2^Department of Medicine and Surgery, University of Parma, Parma 43126, Italy.; ^3^Geriatric-Rehabilitation Department, Azienda Ospedaliero-Universitaria di Parma, Parma 43126, Italy.

**Keywords:** Perioperative neurocognitive dysfunction, dementia, cognitive frailty, aging, dysbiosis, gut-brain axis, intestinal mucosa permeability

## Abstract

Delirium is a clinical syndrome characterized by an acute change in attention, awareness and cognition with fluctuating course, frequently observed in older patients during hospitalization for acute medical illness or after surgery. Its pathogenesis is multifactorial and still not completely understood, but there is general consensus on the fact that it results from the interaction between an underlying predisposition, such as neurodegenerative diseases, and an acute stressor acting as a trigger, such as infection or anesthesia. Alterations in brain insulin sensitivity and metabolic function, increased blood-brain barrier permeability, neurotransmitter imbalances, abnormal microglial activation and neuroinflammation have all been involved in the pathophysiology of delirium. Interestingly, all these mechanisms can be regulated by the gut microbiota, as demonstrated in experimental studies investigating the microbiota-gut-brain axis in dementia. Aging is also associated with profound changes in gut microbiota composition and functions, which can influence several aspects of disease pathophysiology in the host. This review provides an overview of the emerging evidence linking age-related gut microbiota dysbiosis with delirium, opening new perspectives for the microbiota as a possible target of interventions aimed at delirium prevention and treatment.

## INTRODUCTION

Delirium is one of the most frequent conditions complicating the clinical course of patients hospitalized in medical, surgical or intensive care unit (ICU) wards^[1]^. According to a nationwide point prevalence study conducted in Italy some years ago, more than one patient out of five experiences delirium during hospital stay, especially in geriatrics and neurology wards^[2]^. Peaks of delirium incidence have also been detected in critically ill patients admitted to ICU^[3]^, patients under palliative care^[4]^, and geriatric patients with pre-existing dementia^[5]^, where this condition complicates the clinical course in more than 30% of cases. Older patients are generally those with a higher risk of developing delirium^[6]^, but this condition is not exclusive to geriatric patients, being also reported in adults or even children when a critical illness is present^[7,8]^. Furthermore, delirium has been recently recognized as the most frequent complication of severe coronavirus disease-2019 (COVID-19) related pneumonia^[9,10]^.

Delirium has been defined as a severe neuropsychiatric syndrome characterized by an acute change in cognitive functions and acute onset of attention and awareness deficits, with a typically fluctuating course^[1]^. Altered arousal, ranging from reduced responsiveness that may be confused with coma to hypervigilance and severe agitation with aggressive behavior, signs of psychosis, including delusions and hallucinations, and altered mood are frequently present^[1]^. According to the prevalent clinical manifestation, delirium may be classified into three different subtypes: hyperactive, when agitation and aggressiveness prevail; hypoactive, when altered arousal is present; and mixed, when the clinical picture rapidly shifts from hyperactive and hypoactive phases^[1]^. Hypoactive subtype is the commonest one that can be detected in older patients, especially when dementia is present^[11]^.

**Figure 1:**
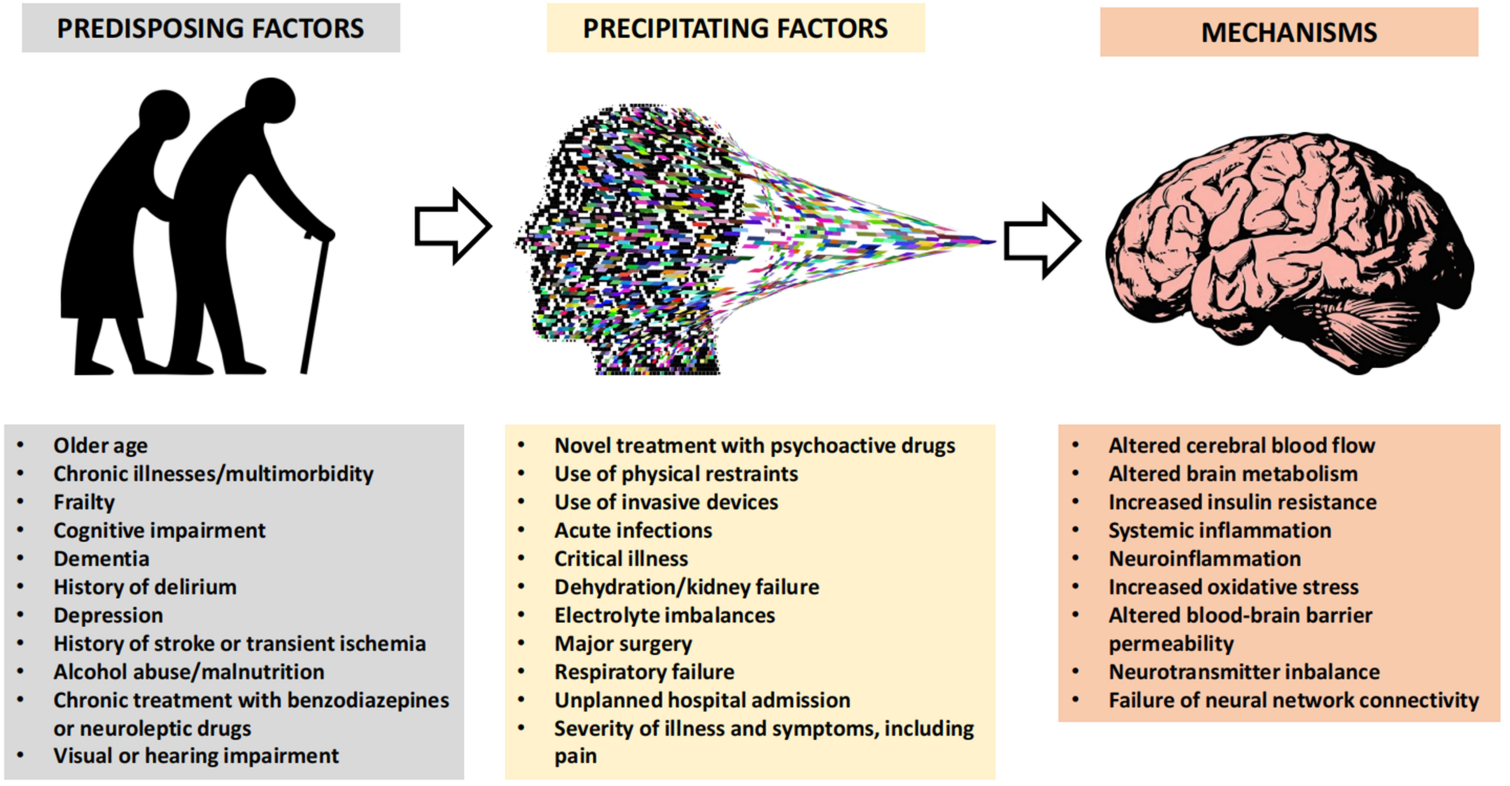
Overview of predisposing factors, precipitating factors and mechanisms involved in the pathophysiology of delirium in patients admitted to hospital for an acute illness.

The pathophysiology of delirium is not completely understood and is generally interpreted as the interaction between acute precipitating factors related to the illness requiring hospitalization, and background predisposing factors prompting brain vulnerability^[12,13]^, highlighted in Figure 1. Systemic inflammation, neuroinflammation, alterations of brain blood flow, increased blood-brain barrier (BBB) permeability, impaired brain insulin sensitivity, and imbalances in neurotransmitter synthesis are the main mechanisms invoked as precipitating the onset of delirium. Conversely, age, prior cognitive impairment, dementia, high comorbidity burden, frailty, malnutrition, and chronic neuroleptic and sedative drug use have been recognized as the most frequent predisposing factors^[1,12,13]^ [Figure 1].

Interestingly, a large number of studies conducted in the last decade have suggested that alterations in the composition and function of intestinal microbiota can be associated with many of the predisposing and precipitating factors of delirium, especially in older age^[14-16]^. Namely, investigations conducted in animal models and, more sporadically, in human beings support the concept that the gut microbiome can modulate the pathophysiology of several neuropsychiatric illnesses, including dementia and mood disorders^[17]^. However, only a few studies have specifically investigated the relationship between microbiome and delirium onset, either in animal models or human beings.

The aim of this review is to summarize the current evidence linking microbiome with pathophysiological determinants of delirium, either as predisposing or precipitating factors, and its mechanisms, identifying the future perspectives of research in this field.

## GUT MICROBIOME AND PREDISPOSING FACTORS OF DELIRIUM

The gut microbiome is the ensemble of bacteria physiologically colonizing the mucosal surface of human gastrointestinal tract with increasing loads and different ecological structures from duodenum to the distal part of the colon^[14]^. The microbiome also represents one of the main components of fecal matter, and the analysis of the composition of fecal microbiota is generally assumed as equivalent to that of intestinal microbiota, although there is no complete overlap^[18]^. Bacteria are not the only microorganisms residing in the gastrointestinal tract of humans in physiological conditions, because also fungi, Archaea, viruses and protozoa can be present, but their role in human health and disease is far less known^[14,18]^. The fecal microbiome is a very complex ecosystem including a large number of genera and species belonging to different bacterial phyla (Firmicutes, Bacteroidetes, Actinobacteria, Verrucomicrobia, and Proteobacteria, among others)^[18]^. Even with this large biodiversity, most of the bacterial taxa of the human microbiome belong just to two phyla: Bacteroidetes and Firmicutes^[19]^. The prevalence of one or the other phylum identifies two distinct enterotypes of the human microbiome^[20]^.

In healthy adult subjects, the composition of intestinal bacterial communities is characterized by a balance between symbiotic bacteria with purported health-promoting activities, and bacteria that may act as opportunistic pathogens^[21]^. The first group includes *Bifidobacterium* spp., *Lactobacillus* spp, *Akkermansia muciniphila,* and *Faecalibacterium prausnitzii*, and all species able to synthetize short-chain fatty acids (SCFAs)^[22-25]^. All these taxa can be involved in modulation of inflammation and immune system activation, promotion of gut mucosal barrier integrity, and regulation of several metabolic functions of the host. SCFAs, in particular, are important mediators promoting insulin sensitivity, adipose tissue catabolism, protein synthesis, and regulation of basal metabolism^[22-25]^. Additionally, opportunistic pathogens are bacterial species that can cause infection and detrimental consequences for the host under specific conditions^[21]^. They mainly belong to the *Enterobacteriaceae* family and can promote disruption of the intestinal mucosal barrier integrity and chronic activation of the inflammatory response^[26]^.

Many of the factors predisposing to the onset of delirium, listed in Figure 1, are associated with alterations of gut microbiota composition and functionality^[27,28]^. Dysbiosis, defined as an imbalance between bacterial taxa with purported health-promoting activity and bacterial taxa promoting detrimental consequences for the host physiology, including opportunistic pathogens^[29]^, can be frequently found in older subjects exhibiting one or more of the predisposing conditions^[27,28]^.

### Aging and microbiota

Gut microbial communities, which remain relatively stable in composition during adulthood, face deep changes in older age^[30]^. Inter-individual variability and uniqueness indexes generally increase, so it is difficult to precisely define a “normal” gut microbiota composition typical of older age^[31,32]^. The usual Bacteroidetes/Firmicutes ratio may be subverted, with decreased representation of *Prevotella* and overgrowth of *Bacteroides*^[33,34]^. This heterogeneity also reflects in the increased representation of bacteria with pro-inflammatory functions, especially opportunistic pathogens belonging to *Enterobacteriaceae*, at the expense of the representation of beneficial bacteria, including *Faecalibacterium prausnitzii* and *Akkermansia*^[35,36]^, leading to reduced capacity of synthetizing important metabolic mediators such as SCFAs^[37]^. Older age is also associated with reduced resilience of gut microbiota to external stressors, so microbial communities get more vulnerable to the detrimental effects of drugs, including antibiotics, illnesses, and unhealthy lifestyles^[38]^.

Interestingly, the gut microbiota of centenarians seems to be less influenced by these age-related changes, with the maintenance of a core microbiome with a relatively high representation of taxa, including *Bifidobacteria* and *Faecalibacterium prausnitzii*, whose metabolic activity may contribute to slowing down the intrinsic mechanisms of aging^[39]^.

### Frailty, multimorbidity and microbiota

Frailty syndrome is a frequent age-related condition defined as a state of vulnerability to stressors resulting from a cumulative decline in many physiological systems^[40]^. Frail persons often exhibit reduced physical and cognitive performance, high susceptibility to acute illnesses, and reduced autonomy in daily activities^[40]^. Frailty is frequently overlapped with multimorbidity, i.e., the coexistence of at least two chronic diseases impairing health status and contributing to generating complex clinical pictures, so that multimorbidity may be considered, according to some theories, as an important part of the frailty syndrome^[41]^.

Frailty and multimorbidity are strongly associated with a significant risk of delirium, according to a recent meta-analysis^[42]^. In geriatric patients, the coexistence of frailty and delirium represents an adverse prognostic factor^[43]^, even though the clinical course of delirium seems worse in subjects without frailty^[44]^. The susceptibility to delirium has thus been interpreted as one of the possible cognitive manifestations of frailty^[45]^.

Frail subjects generally exhibit significant alterations in gut microbiota composition. One population-based study^[46]^ and three studies conducted on nursing home residents^[31,47,48]^ identified the presence of frailty as one of the main drivers of overall gut microbiota composition and biodiversity in older individuals. Furthermore, frailty was significantly associated with gut microbiota composition both at baseline and on follow-up in an intervention study testing the effects of Mediterranean diet on age-related outcomes^[49]^. Frail patients also exhibited reduced representation of *Faecalibacterium prausnitzii* and other SCFA producers, such as *Roseburia*, in three distinct studies of different sizes^[50-52]^. Other studies identified increased representation of *Ruminococcus, Eggerthella, Oscillospira* and *Coprobacillus* as microbial markers of frailty^[53,54]^. In a population-based study conducted on community dwellers over 65 years old, high habitual dietary fiber intake, usual physical activity and increased abundance of taxa belonging to Bifidobacteriales and Clostridiales orders in the fecal microbiota were able to explain 50.1% of differences in physical fitness, supporting a central role for gut microbiome in the phenotypical manifestations of frailty syndrome^[55]^. Overall, these data suggest that frailty is associated with an imbalance between anti-inflammatory bacterial taxa producing beneficial metabolic mediators, such as SCFAs, and pro-inflammatory opportunistic pathogens.

### Dementia, mild cognitive impairment and microbiota

Dementia is considered the main predisposing factor for delirium, and delirium is also associated with a high risk of developing dementia^[56,57]^, so the two clinical entities are often interconnected in a syndrome labeled as delirium superimposed on dementia^[58]^. Mild cognitive impairment, a prodromic form of dementia, also represents a relevant risk factor for delirium, particularly in surgical wards^[59,60]^.

A large number of studies conducted in animal models of dementia or mild cognitive impairment has demonstrated that these conditions are associated with profound degrees of gut microbiota dysbiosis, which may play an active role in conditioning neurodegeneration, neuroinflammation and brain amyloid deposition^[61,62]^. Conversely, a normal gut microbiota composition and functionality may warrant adequate supply to the brain of bioactive compounds with neuroprotective properties, which are derived from microbial biotransformation of non-nutritional substances contained in foods^[63]^.

Unfortunately, human research on the relationship between dementia (or milder cognitive complaints) and gut microbiota is limited to small studies^[64]^, conducted mainly in subjects of Asian ethnicity. Overall, many studies indicate differences in the composition of fecal microbiota between subjects suffering from dementia and healthy controls^[65-73]^. However, the studies aimed at the identification of microbial biomarkers of dementia did not reach solid results, and the bacteria whose representation was associated with dementia were substantially different across studies^[74-76]^. In the largest study conducted to date^[75]^, the fecal microbiota composition of 83 patients with Alzheimer’s disease was compared with that of 125 with mild cognitive impairment and 94 controls with normal cognition. The investigators found that *Lachnospira, Enterobacter, Enterococcus*, and *Klebsiella* were more represented in controls, while Erysipelotrichales, Staphylococcales, *Dorea*, and *Actinomyces* were more represented in subjects with cognitive dysfunction^[75]^. Erysipelotrichales abundance was also significantly correlated with neuropsychological parameters such as the Mini-Mental State Examination test score^[75]^.

In a recent study conducted on 41 demented and 43 cognitively normal subjects from Kazakhstan, the fecal microbiota of patients with dementia was significantly depleted in *Faecalibacterium prausnitzii*^[76]^. Interestingly, *Faecalibacterium prausnitzii* depletion was identified as the main microbial marker of cognitive dysfunction also in another study conducted on 43 subjects with different cognitive performances, and the administration of this strain as probiotic to a group of mouse models of dementia was able to improve cognitive tests^[77]^. Such findings reinforce the assumption that gut microbiota dysbiosis may be part of the pathophysiology of dementia, and that the microbiome-gut-brain axis may play a pivotal role in patients with this condition.

### Depression

The microbiota-gut-brain axis can exert a wide influence on mood disorders, particularly on the course of depression^[17]^. Meanwhile, depression is a well-established predisposing factor for delirium, especially in the surgical context^[78]^. Several studies conducted in adult patients, recently reviewed by Simpson et al., have shown that depression is associated with substantial differences in gut microbiota beta-diversity, with reduction of the expression of *Prevotellaceae* and *Faecalibacterium prausnitzii,* and overgrowth of *Enterobacteriaceae, Eggerthella*, and *Lactobacillus*, among others^[79]^. A recent study also highlighted that, at a functional level, the microbiota of subjects with mild depression is characterized by alterations in proline metabolism that may influence glutamate and gamma-aminobutyrric acid (GABA) in the brain^[80]^.

However, very few human studies were focused on older subjects, so the contribution of gut microbiota to geriatric depression is still unknown. In one trial testing the effects of a probiotic *Bifidobacterium bifidum* and *Bifidobacterium longum* blend in healthy older community dwellers, microbiota modifications were not associated with significant alterations in Geriatric Depression Scale levels^[81]^. The microbiota could be, however, involved in modulating the therapeutical response to levomilacipran administration in older adults with major depressive disorder, as suggested by a recent proof-of-concept study^[82]^.

### Malnutrition

Malnutrition, especially when related to an acute illness where imbalance between energy intake and expenditure is present, is widely recognized as a predisposing factor for delirium^[83]^. This association is even more pronounced in the presence of alcohol addiction, which may also act as a delirium trigger in case of abrupt withdrawal^[84]^.

In a group of older subjects from the TwinsUK Cohort, the presence of malnutrition and poor appetite was associated with reduced species richness and diversity of the gut microbiota, with reduced abundance of several taxa including *Lachnospira* and *Bacteroides*^[85]^. These findings, along with those obtained by Claesson^[31]^, Haran^[47,48]^ and their research groups in nursing home residents, suggest that nutritional status is an important environmental factor shaping gut microbiota in older age. In another study by Fluitman et al., poor appetite in older age was associated with higher abundance of *Lachnospiraceae, Ruminococcaceae* and *Dorea*, while *Blautia* representation was associated with reduced Body Mass Index (BMI)^[86]^. Although the clinical significance of these changes in microbiota composition is still unclear, these results allow the hypothesis that malnutrition and poor appetite are among the major drivers of age-related gut microbiota dysbiosis. Alcohol addiction may enhance these phenomena, since it is associated with profound degrees of disruption of gut microbial community structure even at a young age^[87]^.

### Chronic drug treatments and polypharmacy

Polypharmacy, i.e., chronic treatment with five or more medications, frequently occurs in older patients with multimorbidity due to accumulation of pharmacologic prescriptions for each single chronic illness^[88,89]^. Polypharmacy is associated with an increased burden of adverse events, and delirium represents one of the commonest ones^[90,91]^. This association is emphasized when drugs with anticholinergic activity are involved and interact among themselves and with other drugs^[92]^.

Virtually all pharmacologic treatments can modify the gut microbiota composition and function. In addition to antibiotic and antibacterial therapies^[93]^, this assumption has been well demonstrated for proton-pump inhibitors (PPIs), whose chronic use is associated with increased representation of *Enterococcus, Streptococcus, Staphylococcus* and potentially pathogenic strains of *Escherichia coli*^[94,95]^. Furthermore, studies conducted on animal models and patients with schizophrenia suggest that treatment with antipsychotic drugs, including olanzapine and risperidone, is able to induce profound changes in gut microbiota composition^[96]^, with alteration of the *Firmicutes/Bacteroidetes* ratio and reduced representation of taxa with anti-inflammatory properties such as *Akkermansia* and *Alistipes*^[97-99]^. Interestingly, these drugs are frequently used also in older age to treat behavioral and psychological symptoms of dementia, and represent a relevant predisposing factor and trigger of delirium^[1,12,13]^.

Few studies have specifically evaluated the association between polypharmacy and gut microbiota dysbiosis. In a cross-sectional investigation conducted by our research group^[100]^, the number of chronic drug treatments was independently associated with gut microbiota biodiversity and composition in a group of older patients hospitalized for acute extra-intestinal illness. Interestingly, this association was more pronounced in patients taking antipsychotic drugs^[100]^. The role of polypharmacy as a major determinant of gut microbiota composition was also confirmed in a large study conducted on different population-based cohorts of adult and older subjects, where a high number of drugs was associated with profound changes, including a decrease of *Bifidobacterium*, in microbial ecology pointing towards dysbiosis^[101]^. Interestingly, similar changes were observed also in mouse models of aging after the administration of multiple drugs, and were reversible with deprescribing^[102]^.

## GUT MICROBIOME AND PRECIPITATING FACTORS OF DELIRIUM

The relationship between precipitating factors of delirium, listed in Figure 1, and alterations of gut microbiota composition and function is less established, because of the lack of specific studies on the topic. However, many of the precipitating factors of delirium may be connected to the presence of a critical illness, which is recognized as a possible cause of acute disruption in gut microbiota composition^[103]^.

Critical illness, whatever its origin, can be associated with several pathophysiological processes implying an alteration of the gut microbial community homeostasis, including reduced food intake, intestinal dysmotility, glycemic and electrolyte disturbances, intestinal hypoperfusion, disruption of the intestinal mucus layer, hyperproduction of endogenous mediators such as pro-inflammatory cytokines, catecholamines and opioids^[104]^. Furthermore, clinical interventions aimed at treating the critical condition may also alter gut microbiota (for example, bed rest, artificial feeding, and administration of drugs such as antibiotics, PPIs, catecholamines, sedatives and opioids)^[104]^. Overall, these factors rapidly lead to deep degrees of gut microbiota dysbiosis, with overgrowth of *Enterobacteriaceae, Escherichia coli, Pseudomonas aeruginosa*, and *Klebsiella*^[105,106]^. All these taxa may act as opportunistic pathogens, and the concurrent alteration of gut mucosa permeability can contribute to an increase in the risk of enteric sepsis, which is a frequent complication of the clinical course of critical illness^[107,108]^.

The antibiotic treatments administered in many cases of critical illness are obviously important factors facilitating the acute onset of dysbiosis^[109]^. However, profound dysbiosis has also been observed in patients with trauma-related critical illness^[110]^ or critical illness following major surgery^[111]^, whose treatment does not necessarily imply the administration of antibiotics.

The overgrowth of bacterial taxa involved in inflammation, including *Parabacteroides, Fusobacterium, Enterococcus* and *Bilophila*, can be considered as a marker of the risk of sepsis^[112]^. *Enterococcus* spp abundance in fecal microbiota has been particularly associated with the severity and clinical course of critical forms of COVID-19 pneumonia^[113]^. These microbiota alterations are also accompanied by deep rearrangements in the gut mycobiome and virome, which can also increase the risk of systemic fungal infections^[114]^. Furthermore, these changes in gut microbial ecology are also associated with deep rearrangements of serum and fecal metabolome, reflecting profound changes in bacterial populations also at the functional level^[115]^. The degree of gut microbiota dysbiosis associated with critical illness has also been associated with the risk of death in several studies, underlining the importance of this phenomenon also in clinical terms^[116-119]^.

The few studies specifically focused on older patients admitted to hospital with acute/critical illness have shown, if possible, an even deeper degree of gut microbiota dysbiosis. A study by our research group has demonstrated that older patients admitted with severe illness requiring prolonged hospital stay have an extremely reduced species richness in their fecal microbiota, with consistent reduction of taxa associated with purported health-promoting functions, and blooming of opportunistic pathogens, especially Gram-negative such as *Enterobacteriaceae*^[120]^. These changes were also recently confirmed by another study conducted in a hospital setting, where the authors also detected lower levels of dysbiosis in subjects younger than 60 years old admitted for similar conditions^[121]^.

Abdominal surgery is another frequent trigger of delirium. According to recent studies, the gut microbiota before major surgery may represent a predictor of postoperative complications, including infections, anastomotic leakage, and paralytic ileus ^[122,123]^. A recent study by Liu et al. also highlighted that the presence of a high abundance of Enterobacteriaceae in the gut microbiota of human subjects prior to abdominal surgery for gastric cancer was predictive of the onset of postoperative delirium^[124]^. Furthermore, studies conducted in mice suggest that the administration of general anesthesia and subsequent abdominal surgery are relevant disruptors of the gut microbiome structure^[125-127]^. Such occurrence is independently associated, at least in experimental animal models, with delirium-like behaviors^[125-127]^. Therefore, the analysis of gut microbiota before and after major surgery could represent a promising marker of the risk of delirium in this clinical setting.

**Figure 2:**
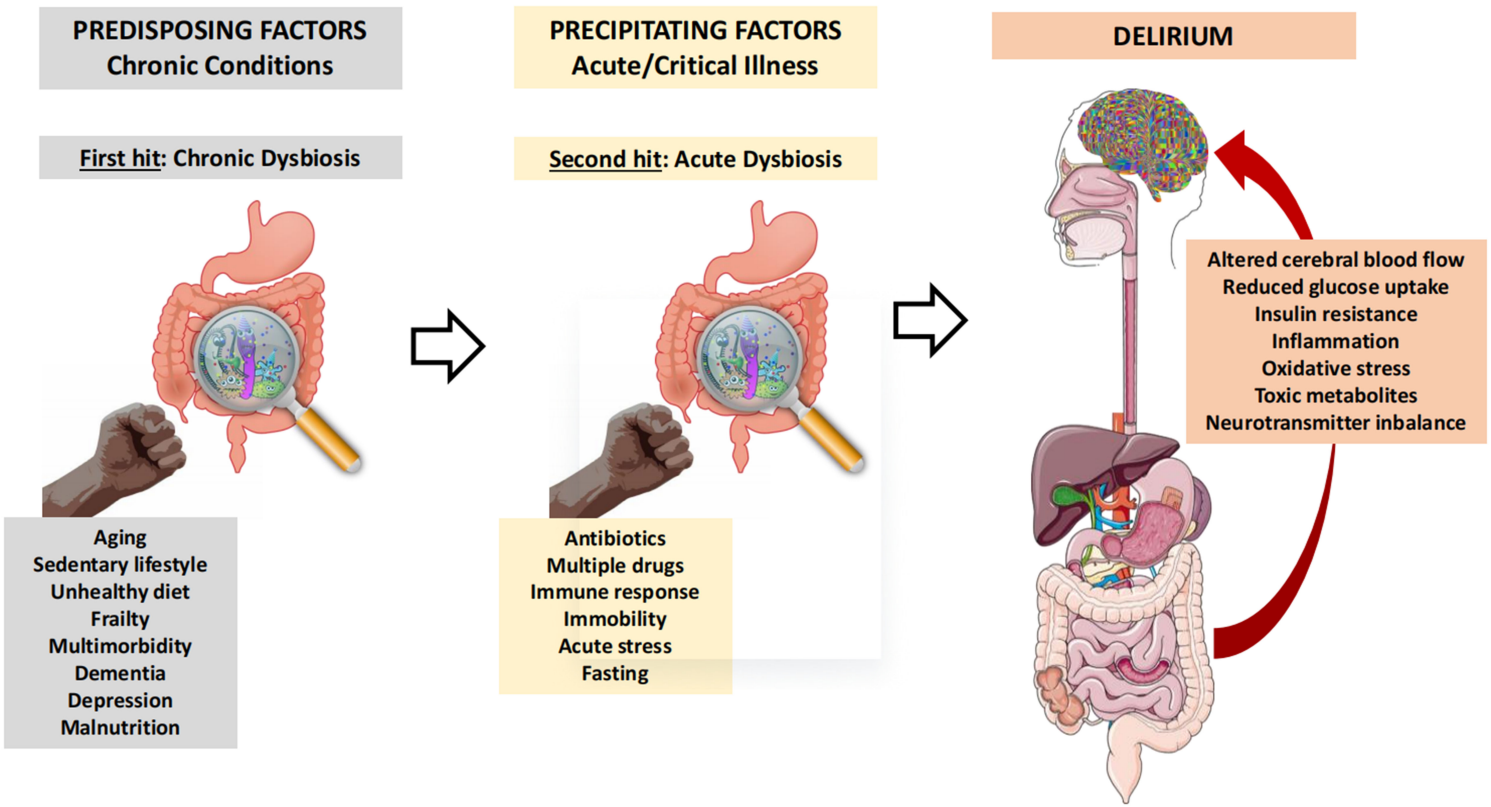
Schematic representation of the pathophysiological model of gut microbiota involvement in the pathogenesis of delirium. Predisposing and precipitating factors of delirium represent two steps leading to acute gut microbiota dysbiosis, which may influence acute cerebral dysfunction through multiple mechanisms.

Therefore, although studies specifically focused on this topic are lacking, the role of gut microbiota in the onset of delirium in hospitalized patients can be hypothesized. In fact, predisposing factors for delirium are largely associated with background gut microbiota dysbiosis, while precipitating factors, especially critical illness, may provide a further hit towards profound disruption of intestinal microbial communities [Figure 2]. These phenomena may have some role in promoting and sustaining the specific pathophysiological mechanisms of delirium.

## GUT MICROBIOME AND MECHANISMS OF DELIRIUM

### Microbiome influence on cerebral blood flow and metabolism

Acute alterations of cerebral blood flow, with reduced oxygen supply to specific areas of the brain, have been recognized as a major mechanism leading to the onset of delirium since the earliest studies^[128]^. In mouse models, microcirculatory and endothelial dysfunction in the brain may lead to diminished oxygenation and alterations of neuronal metabolism^[129]^. These mechanisms have also been confirmed by functional imaging studies in adult septic patients in critical conditions developing delirium during ICU stay^[130-132]^. Furthermore, these acute changes may promote mitochondrial dysfunction in neurons, reducing the capacity of using pyruvate and other substrates for ATP generation^[133,134]^. These mechanisms do not generally lead to the onset of ischemic lesions, but are sufficient to drive major transitory changes in neurotransmission^[135]^.

In mouse models, gut microbiota dysbiosis induced by acute administration of a cocktail of antibiotics was able to promote significant vasoconstriction in middle cerebral arteries, with significant impairment of endothelial nitric oxide synthase (eNOS)^[136]^. In another study conducted in mice, the size of ischemic areas produced by middle cerebral artery occlusion was significantly associated with parameters of gut microbiota dysbiosis, suggesting that the microbiota exerts an important influence on brain microcirculation under stressful conditions^[137]^. Although very preliminary, these findings suggest the existence of a gut-brain axis able to influence the response of cerebral microcirculation to acute injury. The presence of a deep degree of gut microbiota dysbiosis could therefore contribute to the onset of delirium through microcirculatory dysfunction.

Hypoglycemia is known as a risk factor for delirium in diabetic patients and in patients in critical conditions admitted to ICU^[138-140]^. During sepsis, glucose uptake in the brain is substantially reduced even when blood glucose levels are normal, as a consequence of brain insulin insensitivity and reduced representation of the glucose transporter GLUT1 in astrocytes^[141,142]^. All these conditions lead to reduced energy substrate availability for neurons, which may contribute to the development of delirium.

Interestingly, the composition and functionality of the gut microbiota can influence insulin sensitivity, regulating glucose metabolism and utilization in target organs, including the brain^[143]^. Fecal microbiota transplantation is associated with changes in insulin sensitivity that depend on both the characteristics of the microbiota of the donor and the pre-existing functionality of the receiver microbiota^[144-146]^. These changes depend on the synthesis of endocrine mediators by the gut microbiota, particularly SCFAs, such as butyrate and propionate^[147]^. In a recent study conducted in mice, antibiotic-induced gut microbiota dysbiosis was associated with impaired sympathoadrenal response to insulin-induced hypoglycemia^[148]^. The response was restored only after administration of SCFAs, whose synthesis in antibiotic-induced dysbiosis is characteristically impaired^[148]^. Overall, these findings suggest that, in patients with critical illness, gut microbiota dysbiosis may contribute to delirium onset by disrupting the physiological response to hypoglycemia. The resulting impaired glucose utilization in the brain can contribute to acute neuronal dysfunction and impaired neurotransmission, which is the main substrate of delirium.

### Microbiome, inflammation, blood-brain barrier permeability and delirium

Peripheral inflammation and systemic inflammation, such as that occurring during sepsis, are strongly associated with delirium^[149,150]^. Elevated circulating levels of inflammatory biomarkers, including C-reactive protein (CRP) and interleukin-6 (IL-6), represent a risk factor for delirium in hospitalized patients, but clearly, not all patients with pronounced acute inflammatory responses develop delirium^[151-153]^. In animal models, peripheral inflammation represents a trigger of delirium, but the precise mechanisms are not fully understood^[154]^.

In fact, it is unclear how a peripheral phenomenon can influence brain function, especially when BBB is intact. Some evidence suggests that circulating pro-inflammatory cytokines can stimulate the synthesis of inflammatory mediators, such as chemokines and prostaglandins, in cerebral endothelium cells and activate brain perivascular macrophages^[155]^. Other evidence suggests that, in patients with a prior neurodegenerative background, systemic inflammation can trigger microglial activation and neuroinflammation through the mediation of interleukin-1 (IL-1)^[156]^. IL-1 seems able to facilitate chemokine synthesis by astrocytes, which promotes leukocyte homing in the brain tissue, and increase BBB permeability, especially at the choroid plexus level, facilitating the entry of pro-inflammatory mediators in cerebrospinal fluid^[157-159]^. Interestingly, recent evidence suggests that temporary increases in gut mucosal permeability, such as those occurring during acute illness and favored by gut microbiota dysbiosis, may be associated with temporary permeability also at the BBB level^[160,161]^. Such occurrence during a situation of systemic inflammation may imply the entry into the cerebral compartment of pro-inflammatory mediators with potential deliriogenic activity^[160,161]^.

Older subjects with cognitive decline frequently exhibit BBB dysfunction with increased permeability to medium and high-molecular-weight molecules^[162]^. Such occurrence could allow the entry of pro-inflammatory cytokines, such as IL-1 and IL-6, and bacterial products, such as lipopolysaccharide (LPS), into the brain compartment, leading to microglial activation^[163-165]^. Activated microglia can prompt neuronal damage and apoptosis, and impair synaptic remodeling^[166]^. These mechanisms have been well demonstrated in the pathogenesis of Alzheimer’s disease^[163-166]^, but studies focused on delirium are still lacking. However, a certain level of BBB dysfunction and microglial activation with neuroinflammation can also be hypothesized in this setting^[167]^.

Gut microbiota dysbiosis is a well-known trigger of systemic inflammation and, in the context of neurodegenerative disorders, also of neuroinflammation^[168,169]^. Dysbiosis with reduced representation of bacterial taxa able to produce SCFAs, including *Roseburia* and *Faecalibacterium*, and overgrowth of Gram-negative opportunistic pathogens and *Bacteroides* is associated with immune system stimulation and activation of the systemic inflammatory response^[168]^. These changes, in fact, imply increased production of LPS, which can activate Toll-like receptor (TLR) signaling in enteric mucosa cells, which represents a trigger for pro-inflammatory cytokine release^[170]^. LPS can itself overcome the intestinal barrier and enter systemic circulation, where it can activate innate immunity cells^[171,172]^. Bacterial products or even entire bacterial cells can enter systemic circulation in a context of deep dysbiosis with severe gut mucosal barrier impairment, with further stimulation of inflammation and immune response^[173]^. The reduced synthesis of SCFAs is also a trigger for inflammation, because these compounds, particularly butyrate, exert an anti-inflammatory role^[174]^.

### Microbiome, tryptophan metabolism and delirium

The gut microbiota is consistently involved in the metabolism of the amino acid tryptophan in the gastrointestinal tract^[175]^. First, gut bacteria harboring specific functionalities can transform tryptophan into indole and its derivatives^[176,177]^. Indole-producing bacteria may include strains of *Bacteroides*, *Bacillus, Clostridium, Lactobacillus*, and *Escherichia coli*, so their representation is generally high in gut microbial communities^[178]^. In fact, indole and its derivatives are used by many bacterial species as intercellular signaling molecules, regulating several aspects of bacterial physiology, including virulence, biofilm formation and drug resistance^[178,179]^. The virulence of some opportunistic pathogens harbored in the gut microbiota seems to be positively correlated with indole concentrations in the gut lumen^[179]^, but this compound also has a beneficial modulatory effect on the intestinal mucosa, stimulating the production of IL-22 by lamina propria lymphocytes and promoting proliferation of epithelial cells as a mechanism of protection against mucosal damage^[180]^. In particular, the interaction between indole derivatives and aryl hydrocarbon receptors seems critical for preserving mucosal barrier integrity^[181]^. Among the bacterial species able to synthetize indoles, *Bacteroides ovatus* has been recently identified as able to downregulate local intestinal inflammation^[182]^, promote the synthesis of SCFAs and regulate intestinal neurotransmitter release^[183]^. Therefore, *Bacteroides ovatus* may exert an important role in the gut-brain axis^[183]^.

The relevance of these mechanisms for the pathophysiology of delirium is still unclear, but a reduced representation of indole-synthetizing taxa in the context of a severely dysbiotic microbiota may promote activation of the inflammatory response, altered intestinal permeability and stimulation of neuroinflammation in subjects with dysfunctional BBB^[184]^.

Second, tryptophan can be transformed into kynurenine and its derivatives kynurenic acid, quinolinic acid, picolinic acid and xanthurenic acid in the gut immune and epithelial cells^[167]^. The limiting step of this metabolic pathway is the enzyme indoleamine 2,3-dioxygenase (IDO), which is regulated by gut microbiota^[185,186]^. The final products of the kynurenine pathway can be absorbed into circulation and exert modulatory effects on the brain^[187,188]^. Namely, some compounds have pro-excitatory functions on neurons, while others exhibit anti-excitatory properties and promotion of GABAergic transmission^[187,188]^. Kynurenic acid is neurotoxic, and its brain levels have been associated with impaired cognition^[189,190]^. Quinolinic acid increases glutamate activity in the synaptic space and is also associated with impaired memory function^[191,192]^. A recent study conducted in patients admitted to ICUs after surgery has highlighted that the onset of delirium was correlated with increased kynurenic and quinic acid synthesis in the gut^[193]^. Since the microbiota is able to regulate the critical step in the synthesis of these compounds, dysbiosis may contribute to delirium onset also through this complex pathway.

Finally, dietary tryptophan can be transformed into serotonin (5-hydroxytryptamine) in intestinal enterochromaffin cells^[194]^. Germ-free mice exhibit reduced intestinal serotonin synthesis. This circumstance suggests that the microbiota can be consistently involved in intestinal serotonin synthesis, although the precise mechanisms are unknown^[194]^. A role of bacterial SCFAs or deoxycholate produced by bacterial biotransformation of bile acids has been suggested^[194-197]^. SCFAs, particularly acetate, can in fact stimulate serotonin synthesis and promote maintenance of an adequate permeability of gut mucosa^[198,199]^, even if, in experimental models, high acetate loads in the gut lumen, associated with massive serotonin release, caused mucosal damage and promotion of inflammation^[200]^. Recent studies have also identified *Bifidobacterium dentium* as a key species in regulating serotonin synthesis at both gut and brain levels, probably through mediation of acetate^[201,202]^. Interestingly, colonization of intestinal mouse microbiota with *Bifidobacterium dentium* was associated with improvements in repetitive and anxiety-like behaviors typical of an excessive serotoninergic tone^[202]^. Other studies have also suggested that *Escherichia coli, Clostridium sporogenes*, and *Lactobacillus brevis* could have a relevant function in regulating intestinal serotonin synthesis and its interplay with neurotransmission ^[203-205]^. Intestinal serotonin is generally not available to systemic circulation and does not cross the BBB. However, the increased serotonin synthesis in the gut may reduce the bioavailability of tryptophan for absorption by the gut mucosa. Several clinical studies have suggested that delirium is associated with reduced tryptophan levels in serum and in brain tissue^[206-208]^.

### Other mechanisms: oxidative stress, neurotransmitter imbalance and epigenetic regulation

Oxidative stress may be involved in the pathogenesis of delirium, especially after major surgery^[209]^. Experimental evidence from animal models of postoperative delirium suggests that acute cerebral dysfunction may be associated with increased production of reactive oxygen species (ROS), while the administration of drugs reducing ROS production can result in reduced delirium-like behavior^[210]^. Interestingly, in a cohort of patients undergoing cardiac surgery, reduced baseline antioxidant capacity was predictive of postoperative delirium, while increased representation of the soluble receptor for advanced glycation end products was inversely associated with delirium^[211]^. Oxidative damage during surgery was also able to predict incident delirium in the postoperative period^[212,213]^. In older patients, oxidative stress can also be associated with altered circulating levels of amino acids and their ratios, and the ratio between tryptophan and large neutral amino acids was found as predictive of delirium in a group of acutely ill patients over 65 years old^[214]^.

The intestinal microbiota can influence oxidative stress and antioxidant capacity of plasma^[215]^. In germ-free mice, the burden of oxidative stress is generally reduced in comparison with mice harboring the physiological microbiota^[216]^. Conversely, the presence of an adequate representation of *Bifidobacterium* spp. and *Lactobacillus* spp. in the gut microbiota is associated with attenuation of oxidative stress due to their specific antioxidant functionalities^[217-222]^. The administration of taxa belonging to these bacterial genera as probiotics was in fact associated with reduced oxidative stress burden in animal models^[217-221]^, as happened in human beings treated with a functional food specifically stimulating the growth of these bacterial species in the gut microbiota^[222]^.

Microglial cells are consistently influenced by oxidative stress, because the accumulation of gut microbiome-derived oxidative compounds at this level can impair mitochondrial function, reduce ATP synthesis, and lead to global cerebral dysfunction^[216]^. The relevance of these mechanisms has been considered, to date, almost exclusively in the context of neurodegenerative disorders, such as Parkinson’s and Alzheimer’s disease^[223]^, but could also be involved in delirium. Gut microbiota dysbiosis, in fact, is associated with a significant rise in oxidative stress^[224]^. This phenomenon is also dependent on the capacity of the microbiome to transform dietary polyphenols into bioactive compounds with antioxidant properties^[63]^.

The high risk of post-surgical delirium associated with general anesthesia has been mainly associated with neurotransmitter imbalance and direct effects of anesthetic drugs on the brain neural network^[225]^. Interestingly, evidence from animal models suggests that general anesthesia is associated with a high degree of intestinal microbiota dysbiosis, resulting in the failure of the microbiota to produce endocrine and metabolic mediators that are important for the correct function of the gut-brain axis^[225,226]^. In mouse models of surgical stress, gut microbiota dysbiosis was also associated with multiple neurotransmitter system dysfunctions, especially regarding the serotonin and GABAergic systems^[227]^. Although the precise association between gut microbiota dysbiosis and impaired neurotransmission is still unknown, it should be better investigated in the context of delirium, where impaired neurotransmission is frequently found as one of the main pathophysiological mechanisms.

Finally, recent evidence suggests that the gut microbiome is implied in epigenetic regulation of host cells through multiple mechanisms^[228]^. First, several bacterial species, including *Bifidobacterium* and *Lactobacillus*, synthetize significant amounts of folic acid or S-adenosylmethionine, which are fundamental cofactors for DNA methylation^[229]^. Second, microbiota composition seems able to regulate the expression of antibacterial and anti-inflammatory pathways in immune cells and intestinal epithelial cells through the regulation of DNA methylation^[230]^. Ethionine, a microbial metabolite produced by *Lactobacillus reuteri*, seems particularly involved in this regulation^[231]^. The microbiota may also modulate gene expression through histone modifications^[232]^, including deacetylation and lysine crotonylation^[233,234]^. SCFAs could play a pivotal role in these processes, especially in T cells, contributing to downregulating pro-inflammatory cytokine production^[235]^. This issue is of particular relevance in aging, because chronic activation of the inflammatory response, the so-called inflammaging, is linked with the pathogenesis of several chronic illnesses^[236]^. Experimental evidence also suggests that the microbiota composition is able to modulate the expression of long noncoding RNA molecules (lncRNA), which are known for their role in epigenetic regulation^[237]^.

The understanding of the role of epigenetic regulation in the pathophysiology of delirium is still at the beginning. However, recent studies comparing the genome-wide DNA methylation pattern between patients developing delirium after major surgery and patients not suffering from this complication revealed significant differences^[238-240]^. In particular, aging was associated with reduction of DNA methylation involving genes coding for cytokines and other pro-inflammatory factors, especially in leukocytes and microglia^[241]^. These changes were emphasized in patients developing delirium after surgery. Animal studies also suggest an involvement of epigenetic regulation of mitochondrial DNA in the genesis of delirium^[134]^. Therefore, the interaction between gut microbiota, epigenetics, and delirium pathophysiology could represent a promising field of research in the future^[242]^.

## GUT MICROBIOME AND DELIRIUM: THE STUDIES

In the last five years, an increasing number of animal and human studies have focused on the hypothesis that gut microbiota is involved in the pathogenesis of delirium. These studies have been mainly conducted in the context of post-surgical delirium, because the pathophysiological cascade and the precipitating conditions leading to delirium are easier to standardize. Animal models of post-surgical delirium are rather simple to obtain, and the presence of postoperative cognitive dysfunction can be assessed with simple tests such as the Morris water maze, the Y maze, and the buried food test^[126,127,243-246]^.

Zhang et al. first reported that mice developing delirium-like behavior after receiving general anesthesia and surgical laparotomy exhibit a different gut microbiota profile, characterized by a higher degree of dysbiosis, than mice not developing significant post-surgical cognitive dysfunction^[126]^. Interestingly, gut microbiota transplantation from mice without delirium to mice exhibiting delirium-like behaviors was also able to improve cognitive symptoms^[126]^.

All the other studies conducted on animal models of surgical stress included an intervention modifying the gut microbiota composition in their design^[127,243-246]^. Such interventions regarded the administration of prebiotics^[243]^, probiotics, particularly Lactobacilli^[127,244,245]^, and even electroacupunture^[246]^. In all studies, summarized in Table 1, the gut microbiome-centered interventions were able to alleviate post-surgical dysbiosis and were associated with improvement in cognitive delirium-like behaviors^[127,243-246]^. The circumstance that electroacupuncture was able to modify gut microbiota composition also suggests that top-down signaling from the brain to the enteric nervous system may have an important role in the pathophysiology of delirium-associated gut microbiota dysbiosis^[246]^.

**Table 1:** Overview of the existing animal studies focused on the relationship between gut microbiota and delirium (literature updated to February 28th, 2023)

**Authors**	**Setting**	**Study design**	**Participants**	**Main results**
Yang *et al.* 2018^[243]^	Experimental model of abdominal surgery	Intervention (B-GOS supplementation *vs* water)	Adult rats	Improved novel object recognition, increased beta diversity of the microbiome, reduced microglial activation 3 days after surgery
Zhang *et al.* 2018^[126]^	Experimental model of laparotomy	Observational	C57BL/6 mice	Mice developing delirium-like behaviors, assessed through open-field, elevated plus maze and buried food tests, had higher levels of gut microbiota dysbiosis
Jiang *et al.* 2019^[244]^	Experimental model of tibial fracture surgical fixation	Intervention (antibiotic mix or probiotic VSL#3 *vs* water)	C57BL/6 mice	Prevention of decline of the Morris water maze test performance and prevention of post-surgical gut microbiota dysbiosis in mice treated with antibiotics or probiotics
Wen *et al.* 2020^[245]^	Experimental model of splenectomy	Intervention (antibiotic mix, *Lactobacillus* or sodium butyrate *vs* water)	C57BL/6 mice	Administration of *Lactobacillus* and sodium butyrate significantly improved the post-surgical performance on Y maze escape test and prevented the increase of BBB permeability
Liufu *et al.* 2020^[127]^	Experimental model of laparotomy	Intervention (intragastric *Lactobacillus vs* water)	Female mice	Probiotic administration mitigated post-surgical dysbiosis in gut microbiota, inflammation, brain mitochondrial dysfunction and delirium-like behaviors (assessed with Y maze escape test)
Yang *et al.* 2022^[246]^	Experimental model of foot incision-induced surgical pain	Intervention (electroacupuncture stimulation)	C57BL/6 mice	Electroacupuncture ameliorated surgical pain, delirium-like behaviors assessed by open field, Y maze and Buired food tests, and post-surgical gut microbiota dysbiosis

BBB: Blood brain barrier; GOS: galacto-oligosaccharide.

The results of recent studies conducted in human cohorts suggest that the preclinical findings of studies summarized in Table 1 may also have clinical relevance for prevention and treatment of postoperative delirium^[247-252]^. The findings of the human studies investigating the relationship between microbiota and delirium are summarized in Table 2. These studies indicated that the onset of delirium after surgery is associated with specific features of pre-operative or postoperative microbiota composition^[247,248,252]^. In one randomized controlled trial, mechanical bowel preparation procedures before surgery prompted the onset of gut microbiota dysbiosis, which was associated with increased delirium risk after gastrectomy^[249]^. A case report of fecal microbiota transplantation in an oldest-old subject with recurrent *Clostridium difficile* infection also showed that the procedure determined complete recovery of persistent delirium^[250]^. Finally, a recent Mendelian randomization study conducted on very big datasets merging information from multiple cohorts suggested that delirium risk is associated with a specific gut microbiota signature, with increased abundance of *Desulfovibrionaceae* and decreased abundance of several bacterial taxa, including *Oxalobacteriaceae*, *Holdemania*, *Ruminococcus*, and *Eggerthella*^[251]^.

**Table 2:** Overview of the existing human studies focused on the relationship between gut microbiota and delirium (literature updated to February 28th, 2023)

**Authors**	**Setting**	**Study design**	**Participants**	**Main results**
Maekawa *et al.* 2020^[247]^	Patients undergoing cardiac surgery	Observational, longitudinal	21 patients (age median 62, range 22-80 years old)	Gut microbiota biodiversity was reduced after surgery, and the abundance of *Pseudomonas* and *Staphylococcus* positively correlated with pseudopsia and delirium
Liu, *et al.* 2022^[248]^	Patients undergoing gastrectomy for cancer	Observational, longitudinal	40 patients aged ≥ 65 years old (mean 70)	Gut microbiota composition before surgery was predictive of delirium (several taxa positively correlated with delirium, including *Escherichia/Shigella, Klebsiella, Ruminococcus,* and *Lactobacillus*)
Yang, *et al.* 2022^[249]^	Patients undergoing gastrectomy for cancer	Intervention, RCT (MBP *vs* no MBP)	81 patients aged ≥ 65 years old (mean 73)	MBP was associated with significant differences in gut microbiota composition and delirium risk. The abundance of *Bacteroides* and *Veillonella* was associated with delirium, while *Olsenella* was protective
Gotoh, *et al.* 2022^[250]^	One patient with *Clostridium difficile* infection	Intervention (FMT)	One 92-year-old woman	FMT determined a dramatic increase in fecal microbiota biodiversity and was associated with recovery from persistent delirium
Yu, *et al.* 2023^[251]^	Genetic data from GWAS conducted in 24 cohorts	Mendelian randomization study	18,340 individuals from 24 cohorts	Genetic prediction of the family *Desulfovibrinaceae* was associated with increased genetically predicted delirium risk*Oxalobacteriaceae, Holdemania, Ruminococcus*, and *Eggerthella* was protective against genetically predicted delirium risk
Xie, *et al.* 2023^[252]^	Patients undergoing elective orthopedic surgery	Observational, longitudinal	86 patients (median age 71 years old)	The gut microbiota abundance of *Parabacteroides diastasonis* in postoperative fecal microbiota was positively associated with incident delirium

FMT: Fecal microbiota transplantation; GWAS: genome-wide association study; MBP: mechanical bowel preparation; RCT: randomized controlled trial.

Unfortunately, no intervention studies with probiotics or functional foods in older subjects have investigated delirium as an endpoint to date^[252]^. However, bioactive substances resulting from the interaction between gut microbiota and dietary polyphenols have a great potential to modulate brain function and counteract several of the pathophysiological mechanisms of delirium^[63,253,254]^. The administration of polyphenolic nutraceutical supplements to animal models of neurodegenerative diseases has produced very promising results^[253]^, and preliminary evidence from human studies also suggests that flavonoid supplementation could be effective in attenuating cognitive symptoms, especially in the context of the long COVID-19 disease^[254]^. A recent human study has shown that habitual consumption of tea, a beverage naturally rich in phenolic compounds, was associated with a markedly lower incidence of postoperative delirium after elective orthopedic surgery^[255]^. The role of nutrition and traditional medicine in modulating gut microbiota towards protection against the onset of delirium may therefore represent another promising field of research for the future.

In summary, the current state-of-the-art of scientific literature supports the assumption that gut microbiota alterations are involved in the pathophysiology of delirium, especially in the postoperative setting. However, to date, the state of knowledge is still at the beginning and the clinical implications of this putative association are unknown. Furthermore, very few studies were specifically focused on the characteristics of older patients with frailty and multimorbidity, who are the ideal subjects at risk for developing delirium during hospital admission.

## CONCLUSIONS

Both predisposing and precipitating factors of delirium are associated with alterations in gut microbiota composition and functionality, pointing towards dysbiosis. The gut microbiota is also potentially involved in the regulation of pathophysiological mechanisms leading to the onset of delirium, including neuroinflammation and alterations of the BBB permeability. Both animal and human studies suggest that intestinal microbiota dysbiosis is associated with an increased risk of delirium in the post-surgical setting, and that this association could be mitigated by the administration of prebiotics or probiotics, at least in animal models. The relationship between gut microbiota and delirium represents a promising area of research bridging together gerontology, microbiology, and clinical sciences.

## DECLARATIONS

### Copyright

© The Author(s) 2023.
